# Quantitative Variation of Flavonoids and Diterpenes in Leaves and Stems of *Cistus ladanifer* L. at Different Ages

**DOI:** 10.3390/molecules21030275

**Published:** 2016-02-27

**Authors:** Cristina Valares Masa, Teresa Sosa Díaz, Juan Carlos Alías Gallego, Natividad Chaves Lobón

**Affiliations:** Department of Plant Biology, Ecology and Earth Sciences, Faculty of Science, University of Extremadura, 06006 Badajoz, Spain; cvalmas@unex.es (C.V.M.); jalias@unex.es (J.C.A.G.); natchalo@unex.es (N.C.L.)

**Keywords:** *Cistus ladanifer* L., phenols, flavonoids, diterpenes, age, quantitative variation

## Abstract

The compounds derived from secondary metabolism in plants perform a variety of ecological functions, providing the plant with resistance to biotic and abiotic factors. The basal levels of these metabolites for each organ, tissue or cell type depend on the development stage of the plant and they may be modified as a response to biotic and/or abiotic stress. As a consequence, the resistance state of a plant may vary in space and time. The secondary metabolites of *Cistus ladanifer* have been quantified in leaves and stems throughout autumn, winter, spring and summer, and at different ages of the plant. This study shows that there are significant differences between young leaves, mature leaves and stems, and between individuals of different ages. Young leaves show significantly greater synthesis of flavonoids and diterpenes than mature leaves and stems, with a clear seasonal variation, and the differences between leaves at different growth stages and stems is maintained during the quantified seasons. With respect to age, specimens under one year of age secreted significantly lower amounts of compounds. The variation in the composition of secondary metabolites between different parts of the plant, the season and the variations in age may determine the interactions of *Cistus ladanifer* with the biotic and abiotic factors to which it is exposed.

## 1. Introduction

Plants have developed diverse strategies for protection against different stressful factors; on the one hand, the development of structures like thorns, glandular hairs and foliar trichomes [1,2] and, on the other hand, the production of secondary metabolites. The latter have a variety of roles in the life of plants: they act as defence against predators and pathogens, acting as deterrents and inhibitors of feeding and oviposition [3,4,5], inhibiting insect growth and development [6,7], as allelopathic agents [8,9,10], enhancing the attraction of pollinators [11,12], resistance to viruses [13], protection against ultraviolet radiation [14,15], even acting as signal molecules for nodulation in legume-*Rhizobium* symbiosis [12,16] and as antioxidant agents [17,18].

Secondary metabolites vary qualitatively and quantitatively between different species and also between populations of the same species, between individuals of those populations and between the organs of a certain individual, to such an extent that some authors defend phenolic quantification as an effective method to assess the level of chemical defence of a plant [19,20,21,22,23].

*Cistus ladanifer* L. (rock-rose or “jara”) is a Mediterranean shrub [24,25] that is widely distributed over western Iberia and northern Morocco [26], growing under very diverse and extreme climates and standing cold stress, dryness and high temperatures [27]. Through its leaves and photosynthetic stems, this species secretes an exudate that has been studied by different authors [28], mainly due to its interest in the perfume, pharmaceutical and food industries [29,30].

**Figure 1:**
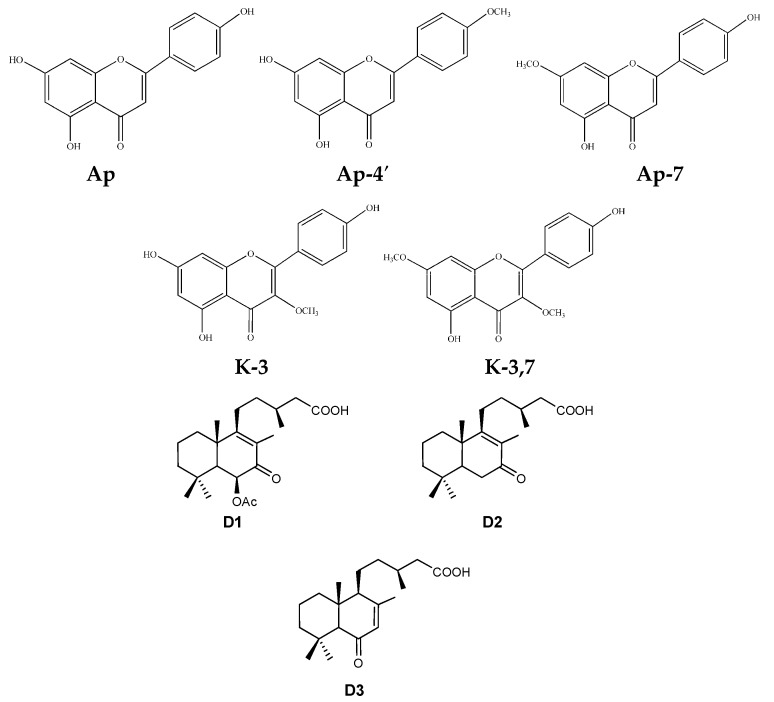
The chemical structures of flavonoids and diterpenes in the exudate of *Cistus ladanifer*. **Ap**: apigenin; **Ap-4′**: apigenin 4′-methyl ether; **Ap-7**: apigenin 7-methyl ether; **K-3**: kaempferol 3-methyl ether; **K-3.7**: kaempferol 3,7-di-*O*-methyl ether **D1**: 6-acetoxy-7-oxo-8-labden-15-oic acid; **D2**: 7-oxo-8-labden-15-oic acid; **D3**: oxocativic acid.

The exudate of *C. ladanifer* is composed fundamentally of compounds of phenolic and terpene origin [31,32,33,34,35,36,37]. In previous studies it has been shown that the phenolic compounds synthesized by *C. ladanifer*, in particular aglycone flavonoids (apigenin (Ap), kaempferol 3-methyl ether (K-3), apigenin 4′-methyl ether (Ap-4′), apigenin 7-methyl ether (Ap-7) and kaempferol 3,7-di-*O*-methyl ether (K-3.7)) constitute between 6% and 26%, of the dry weight of the exudate, depending on the season [38]. Their synthesis is markedly seasonal: they are the majority products in summer, but in winter their presence is at a minimum [34]. Studies quantifying the terpenes have found that the concentrations in the exudate of *C. ladanifer* are generally in the range of 1%–2% dry weight of the exudate [35] and that the majority terpenes are three diterpenes (6-acetoxy-7-oxo-8-labden-15-oic acid (D1), 7-oxo-8-labden-15-oic acid (D2), oxocativic acid (D3)). Figure 1 shows the molecular structures of these compounds (flavonoids and diterpenes).

In previous studies [39,40] we have shown that the synthesis of secondary metabolites in *Cistus ladanifer* L. varies and is induced by ecological factors such as UV-light, hydric stress and temperature. This compounds have been shown to function as UV filters [39,41], as protectors against herbivory [38], and as allelopathic agents [8,9,10,42]. Moreover, the aqueous extract of *C. ladanifer* was able to generate antioxidante, antibacterial and antifungal activities in a dose-dependent manner [43,44].

For all that and to further contribute to a better understanding of the capacity of *C. ladanifer* to respond to different environmental conditions and therefore to the relative resistance state of this shrub, the main objective of the present study is to determine how the chemical profile changes at different ages of the plant, between seasons and between organs (leaves in different stages of development and stems). The results may be helpful in illustrating the importance of secondary compounds in *C. ladanifer* against biotic and abiotic factors.

## 2. Results

### 2.1. Quantitative Variation of Flavonoids and Diterpenes between Young Leaves, Mature Leaves and Stems

**Figure 2:**
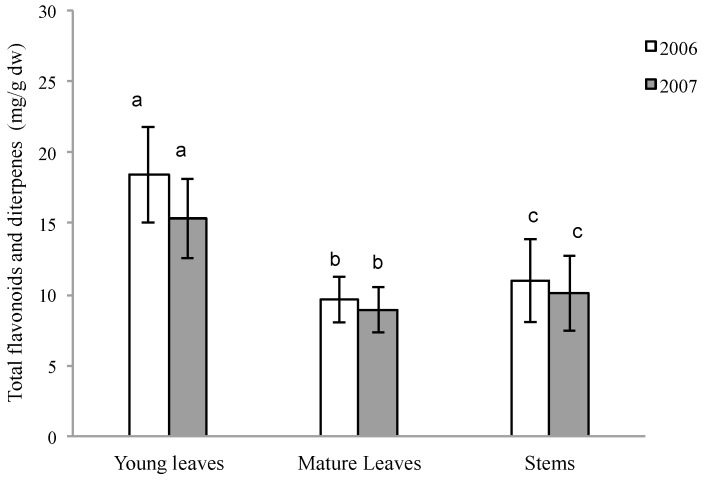
Total flavonoid and diterpene concentrations (mg/g dw) of samples of young and mature leaves and stems within each year of *C. ladanifer*. Error bars, means, standard deviation. Annual mean (*n* = 20 individuals × 3 replicates × 8 seasons = 480). a, b, c: different letters mean significant differences between young and mature leaves and steams *p* < 0.05 (Wilcoxon Test).

**Figure 3:**
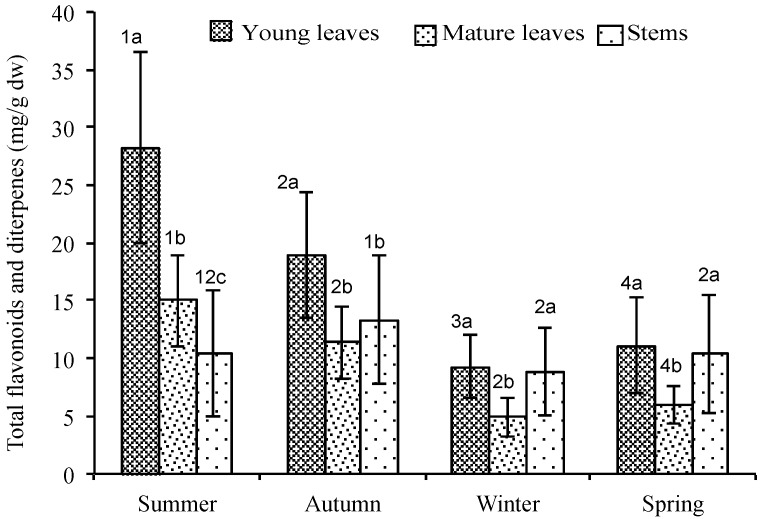
Total flavonoids and diterpenes of the exudate of 20 individuals of *C. ladanifer* in the different tissues analysed, by season and in the different season analysed, by tissues (mg/g dw). Error bars, means, standard deviation. Mean of the two years (*n* = 20 individuals × 3 replicates × 2 years = 120). 1 2, 3, 4: different numbers mean significant differences between seasons *p* < 0.05 (Wilcoxon Test). ^a, b, c^: different letters mean significant differences between young and mature leaves and steams *p* < 0.05 (Wilcoxon Test).

The results demonstrate that the type of organ and its development state are determining factors to quantify these compounds in *C. ladanifer* (Figure 2), being the young leaves the organs that show greater amount, followed by stems. This behaviour was observed throughout the two-year study period, and no significant differences were found between the two years. On the other hand, the amount of compounds derived from secondary metabolism in *C. ladanifer* is dependent on the season, being summer the period in which secretion is significantly greater (Figure 3). It is important to highlight that the amount of secondary metabolites in young leaves is greater than that in mature leaves and stems in all four seasons (Figure 3). The amount of these compounds within the plant and throughout the year shows very high variability, ranging from 28.21 mg/g dw in young leaves during the summer, to 4.93 mg/g dw in mature leaves during the winter.

If we analyse the two families of compounds separately (total flavonoids and total diterpenes) it can be observed that most of the compounds are flavonoids, which represent the majority in all seasons and organs (Table 1). The major flavonoid is K-3.7 and its synthesis is clearly seasonal in both young and mature leaves, showing greater amounts in the summer. The highest secretion in stems takes place in summer-autumn and the lowest secretion during winter-spring. The amount of this compound in the plant varies throughout the year from 2.91 mg/g dw in mature leaves in the winter, to 20.72 mg/g dw in young leaves in the summer. With respect to diterpenes, the largest amounts were quantified in autumn-winter. The most abundant is D1, being its synthesis significantly different between young leaves, mature leaves and stems throughout the whole year. The amount of D1 present in the plant varies throughout the year from 0.42 mg/g dw in mature leaves in the winter, to 3.03 mg/g dw in young leaves in the autumn. It is important to highlight that the greatest amounts of K-3.7 and D1 are shown by young leaves in all four seasons (Table 1).

### 2.2. Quantitative Variation of Flavonoids and Diterpenes between Ages

**Figure 4:**
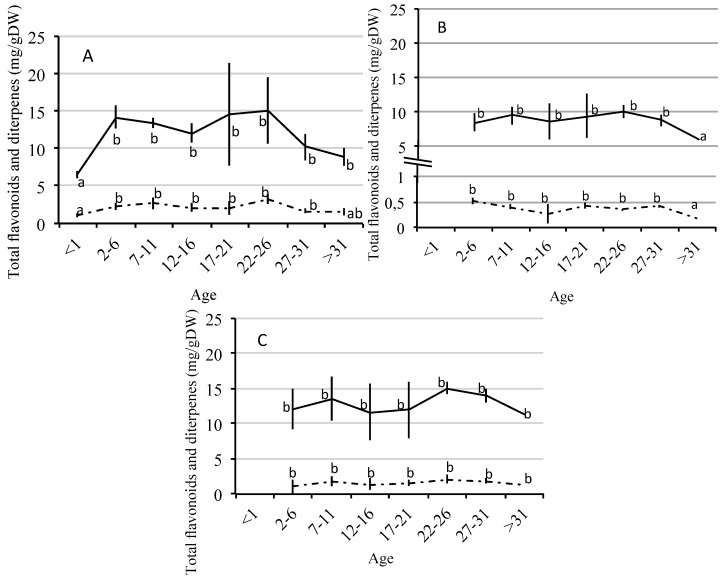
Total flavonoids (—) and diterpenes (·-·-·-·) (mg/g dw) for different age groups in samples of young leaves (**A**); mature leaves (**B**) and stems (**C**). a, b: different letters mean significant differences between ages *p* < 0.05 (Wilcoxon Test).

After quantifying the amount of flavonoids and diterpenes in young leaves, mature leaves and stems from plants of different ages (Table 2; Figure 4), it was observed that, regardless of age, the amount of compounds (both flavonoids and diterpenes) was lower in mature leaves. The individuals under one year of age have only young leaves, and showed lower amounts of both flavonoids and diterpenes than those observed in young leaves from older plants. In the more senescent individuals, the amount of these compounds goes back to lower levels, although these amounts are not significant with respect to the amounts present in these leaves at earlier ages.

Mature leaves are present in individuals over two years of age and the synthesis of flavonoids and diterpenes in these organs shows a significant decrease in senescent individuals (>31 years). In stems, the synthesis of these compounds is less variable among individuals of different ages. In fact, no significant differences were found for the total amount of flavonoids and diterpenes at any age.

## 3. Discussion

The present results have contributed to broadening the understanding of the secondary metabolism of *C. ladanifer*. First of all, it is demonstrated that there are significant quantitative differences of these compounds in the different organs studied. Young leaves secrete greater amounts of secondary metabolites than stems, and these in turn more than mature leaves. Phenols (flavonoids) are the most abundant compounds, being K-3.7 the major flavonoid which is secreted in significantly different amounts in all the plant parts analysed. Studies performed by Del Valle *et al.* [23] show that the accumulation of flavonoids in *Silene littorea* was highly variable among organs within individual plants. These results were supported by the proposal of Davies *et al.* [45], which states that the biosynthesis of flavonoids can be tissue-specifically regulated.

These quantitative differences among tissues are not limited to flavonoids. In *C. ladanifer*, the amount of diterpenes is significantly different among tissues. Moreover, studies carried out with *Rosmarinus officinalis* [46] show this behaviour for diterpenes present in this species.

Characteristics of the plant such as water, protein, and secondary metabolite contents usually change during the development of an organ [47]. The observed existence of greater amounts of the latter compounds in young leaves than in mature leaves could be due to greater secretion in the early stages of growth, and also because the secretion-to-degradation ratio decreases as the leaf ages. For example, some microbes can degrade these compounds [48,49] after secretion. Furthermore, studies of *Empetrum hermaphroditum*, *Betula pendula* and *Hypericum origanofolium* have shown a major decrease in secondary metabolite concentration during leaf senescence [50,51,52,53]. In *Empetrum* *hermaphroditum* only 33% of the total phenols present in young leaves are found in mature leaves, and the proportion of compounds from a development state to another varies depending on the compound. This fact is also demonstrated in *Quercus robur*; studies performed by Covelo and Gallardo [54] showed that the concentrations of phenols from young to mature leaves decreased 37%. In *C. ladanifer* mature leaves show 40% less phenols than young leaves and 45% less in total compounds.

Other studies suggest that in young leaves lacking epidermis, trichomes and their exudates may serve as a functional analogue of the epidermis in mature leaves [55] since they play a similar protective role against biotic and abiotic factors such as water deficit [56,57], insect herbivores [58,59,60], phytopathogenic fungi [61], and UV-B radiation [62,63,64]. At later stages of leaf development, when the formation of the epidermis is completed, the functional role of the trichomes becomes less important, and they often senesce and shed. In some cases, however, trichomes remain viable and functional in mature leaves [65]. Moreover, the composition of exudates produced by glandular trichomes may change with leaf age [66,67]. In our study case, it is demonstrated that mature leaves contain the same compounds as those found in young leaves, although in lower amounts. This fact may involve important consequences for the adaptation of rockrose to different factors.

Secondly, our results show a clear seasonal variation of the secondary metabolites in all the plant parts analysed, although this variation is more leveled in stems. This confirms the observations of previous studies performed in young leaves of *C. ladanifer* for both flavonoids and diterpenes [35,41,68].

If a plant varies the composition of secondary metabolites seasonally and between organs, its resistance level may change throughout the year and depending on the organ. In studies performed by Riipi *et al.* [69] it was demonstrated that the variation in the content of secondary metabolites in leaves of *Betula pubescens* governed the resistance state of this plant to environmental factors; this author asserts that the resistance state of a plant is not constant in time and, thereby, it is important to study the temporal and spatial dynamics of these compounds and the evolutionary implications of their variations [69]. Therefore, metabolic profiles may predict the resistance levels of a plant against different environmental factors [70,71,72] and their analysis could help characterising the species against these factors [73] as shown in studies performed by [74] where a positive correlation was found between the content of flavonoid glycosides and the resistance to certain herbivores.

In previous studies, we have shown that the flavonoids in the exudate of the leaves of *Cistus ladanifer* play an important ecophysiological role. They protect the plant against ultraviolet radiation and have antiherbivory activity, specially K-3.7 [9,10,38]. This compound clearly shows a seasonal and between-organs variation, showing its maximum concentrations in young leaves in the summer. The greatest amount of this compound in this season and in leaves (with greater physiological activity) may contribute to the resistance to UV radiation, pathogens and the attack from herbivore insects, since it is during this season when ultraviolet radiation is highest and herbivore insects are most abundant. The flavonoids that accumulate in the epidermis of leaves of *C. ladanifer* would mitigate the effects of solar radiation by reducing the amount of photosynthetically active radiation transmitted to chlorenchyma [75,76] and to protect against UV-B damage to DNA [77].

Moreover, if we take into account that a plant under stressful conditions (high temperature, strong hydric stress, abundance of herbivore insects, high ultraviolet radiation…) produces high concentrations of free radicals and the damages these involve [78,79,80,81,82], the fact that K-3.7 shows antioxidant activity [18] could explain why the maximum concentration of this compound occurs in the summer season. The ability to regulate the flavonoid accumulation in photosynthetic organs of *C. ladanifer* may represent an advantage for the species in climate change scenarios, where an increase of temperature and UV-B radiation is expected [83].

Diterpenes play diverse functional roles in plants, acting as hormones, regulators of wound- induced responses and antioxidants [84]. *In vivo* studies have shown that the diterpene carnosic acid may protect biological membranes from oxidative damage [85], and that under drought- and high light-induced oxidative stress conditions, the amounts of diterpenes in rosemary leaves increase [86,87]. In *C. ladanifer*, the greatest amount of diterpenes are synthesised in young leaves, although not during the summer, when the conditions of water stress and high temperatures are more harmful. Autumn and winter are the season when maximum amounts of diterpenes are quantified. Thereby, these compounds could be playing a role different from that of antioxidant agents. For instance, diterpenes are also suspected to provide cell membrane stability.

Finally, another important factor to be considered in the quantification of a species’ secondary metabolism is the age of the individuals. The secondary metabolites may change significantly both qualitatively and quantitatively during plant growth and ontogeny. Generally, juvenile stages express stronger defences than mature stages [88] and this may allow them to adapt to different kinds of stress. However, the present results showed that the youngest individuals (under one year of age) secreted significantly less total compounds than older individuals in young leaves. Furthermore, from the following age group of 2-to-6-years onwards, the secretion of compounds remained more or less constant until the last age group considered (above 31 years of age), when the secretion declined again, being this drop significant in mature leaves. This pattern was observed in flavonoids. In these studies performed with *Pyracantha coccinea* [89] flavonoid accumulation during the plant life, shows that the capability of biosynthesising these metabolites appears gradually and, like in *C. ladanifer*, the lowest amounts of flavonoids are shown by the individuals under one year of age.

The results of this study indicate that the synthesis of secondary metabolites in *C. ladanifer* is variable and it depends mainly on the organ and season. The time-space variability in the production of secondary metabolites could affect the ecological interactions of the species and its ecophysiological behaviour, which varies depending on the season and the plant organ.

## 4. Experimental Section

### 4.1. Sample Collection

For the quantification of secondary metabolites in leaves and stems, 20 individuals of 20 mm in trunk base diameter (approximately 12 years of age according to the equation y = 1.5496x + 1.5342R^2^ = 0.9221 [90]) were selected from a stand of the species (“jaral”) in Alburquerque, in the north-west of the Province of Badajoz (SW Spain, 39°08′05.1″ N–7°00′40.54″ O). Table 3 lists the climatic characteristic (rainfall and maximum and minimum temperaturas) for this location [91,92].

At the end of each season, three types of samples were taken from each individual: young leaves (sprouts at the apical part), mature leaves (approximately 6 months of age) and photosynthetic stems (*i.e.*, the apex that had grown during the study year, with a diameter of 1.15–2.25 mm). The samples were individually packed *in situ*, numbered and then stored in bags until they were analysed later in the laboratory (the same day as the sampling). Samples were collected seasonally over two consecutive years.

In order to quantify the age-dependent variability in the composition of secondary metabolites, 110 plants from the rock-rose population previously described were selected on the basis of their different height and stem diameter. Height was measured using a measuring tape and stem diameter using a caliper at the base. Five samples of leaves and photosynthetic stems were collected from each plant and stored at −18 °C. Then, for ring counting, the trunk of each individual was cut at 5 cm from the ground.

### 4.2. Age Determination

Plant age was determined by counting the annual growth rings. A ring per year was assumed, following the pattern observed in temperate zones [93]. To this end, sections of stem brought to the laboratory from the field work were mounted on wooden supports and sanded down. The sanded surface was wetted, and the growth rings counted under a magnifying glass. For each specimen, counts were made by at least 5 people, the extreme values (above and below) were discarded and the mean value was taken.

Once the age of each of the 110 individuals was determined, three individuals of each age range were selected and the samples of young leaves, mature leaves and stems from those individuals were analysed.

In order to quantify the compounds by age, the data of the individuals were binned into age groups in series of 5 years each and the means were taken for the different organs studied.

### 4.3. Extraction and Assay of Secondary Metabolites

Approximately 1 g wet weight of young and mature leaves and stems were taken in three replicates per plant and sample type. The exudate was extracted with chloroform at a ratio of 1:10 *w*/*v* to ensure complete extraction of flavonoids and diterpenes [34,41,94,95,96]. The chloroform was evaporated off under a fume hood at a temperature not exceeding 30 °C and the pellet was re-suspended in 3 mL of methanol. The resulting solution was kept frozen at −20 °C for 12 h to precipitate out the waxes which were then removed by centrifugation and the supernatant was stored at 4 °C for its subsequent analysis [33,97].

The assay of secondary metabolites was carried out by HPLC (Waters, Cerdanyola del Vallès, Spain; Pumps: 515 HPLC Pump; 717-plus Autosampler Injector; 996 Photodiode Array Detector). 80 µL of each sample were injected into a Spherisorb C-18 5 µ 4.6 × 250 mm reverse phase analytical column. The mobile phase used was water/methanol/tetrahydrofuran in the proportion 56/16/28 at a 0.75 mL/min flow rate. The chromatograms were recorded at a maximum wavelength of 350 nm for flavonoids and 250 nm for diterpenes. These conditions yield a chromatogram with optimal resolution for the identification of the five flavonoids [34,41,96] and three diterpenes [35,68] present in the exudate of *C. ladanifer*. The compounds were identified on the basis of their retention times and spectral characteristics [34,41,96]. The detection of flavonoids and diterpenes and the determination of the linear calibration equation were carried out as described in [98]. Finally, the results obtained were expressed with reference to the leave or stem dry weight.

### 4.4. Statistical Analyses

The data were non-normally distributed; therefore, non-parametric statistical tests were applied. In particular, the results were first submitted to the Friedman test (SPSS-Win 15.0 SPSS Inc., Chicago, IL, USA) to determine whether there were significant differences between seasons, organs and ages, and then the Wilcoxon test for paired samples (SPSS-Win 15.0) was applied to establish between which seasons, organs and ages there were significant differences. Differences were considered significant at a level of *p* ≤ 0.05.

## Figures and Tables

**Table 1:** Total flavonoids and diterpenes (standard deviation) of the exudate of 20 individuals of *C. ladanifer* in the different tissues analysed, by season (mg/g dw). Mean of two years.

Compounds	Summer			Autumn			Winter			Spring		
	Young Leaves	Mature Leaves	Stems	Young Leaves	Mature Leaves	Stems	Young Leaves	Mature Leaves	Stems	Young Leaves	Mature Leaves	Stems
**Flavonoids**												
**Ap**	0.12(0.04) a	0.08(0.03) b	0.11(0.06) a,b	0.15(0.11) a	0.07(0.02) b	0.16(0.07) a	0.10(0.05) a	0.03(0.01) b	0.11(0.06) a	0.08(0.04) a	0.05(0.02) b	0.15(0.15) a
**K-3**	1.95(0.95) a	1.09(0.51) b	1.25(0.86) c	1.66(1.08) a	0.91(0.46) b	2.02(0.96) c	0.75(0.53) a	0.27(0.20) b	0.80(0.52) a	0.83(0.45) a	0.32(0.16) b	1.13(0.90) c
**Ap-4′**	1.37(0.28) a	0.78(0.19) b	1.06(0.43) c	2.06(1.03) a	0.69(0.16) b	1.30(0.66) c	1.28(0.25) a	0.56(0.11) b	1.48(0.36) c	1.01(0.42) a	0.75(0.25) b	1.79(0.81) c
**Ap-7**	2.49(0.58) a	1.41(0.24) b	1.42(0.54) b	2.56(1.12) a	1.21(0.51) b	1.68(0.69) c	1.46(0.33) a	0.67(0.13) b	1.40(0.64) a	1.43(0.49) a	1.07(0.38) b	1.64(0.75) c
**K-3.7**	20.72(6.62) a	10.82(3.30) b	6.68(3.50) c	9.25(4.01) a	7.85(2.42) a	7.46(3.40) a	3.22(1.64) a	2.91(1.29) b	3.64(2.29) c	5.95(2.66) a	3.27(1.09) b	4.48(3.59) c
Total	26.65(7.90) a	13.99(3.86) b	9.90(5.25) c	15.59(5.09) a	10,71(2.99) b	12.58(5.29) a,b	6.78(2.10) a	4.45(1.59) b	7.51(3.05) a	9.29(3.67) a	5.45(1.41) b	9.18(4.85) a
**Diterpenes**												
**D1**	1.44(0.43) a	0.75(0.24) b	0.53(0.26) c	3.03(1.61) a	0.48(0.19) b	0.66(0.40) c	2.14(1.03) a	0.42(0.33) b	1.21(1.45) c	1.56(0.62) a	0.57(0.32) b	1.15(0.60) c
**D2**	0.08(0.03) a	0.04(0.03) b	0.02(0.01) c	0.17(0.16) a	0.02(0.01) b	0.03(0.01) b	0.12(0.07) a	0.02(0.01) b	0.06(0.05) c	0.09(0.03) a	0.03(0.02) b	0.05(0.03) b
**D3**	0.04(0.02) a	0.02(0.02) b	0.02(0.02) b	0.13(0.06) a	0.02(0.01) b	0.04(0.02) c	0.35(0.28) a	0.05(0.04) b	0.13(0.10) c	0.09(0.04) a	0.03(0.02) b	0.07(0.05) c
Total	1.56(0.46) a	0.81(0.26) b	0.56(0.30) c	3.32(1.75) a	0.52(0.20) b	0.72(0.43) c	2.60(1.11) a	0.48(0.36) b	1.46(1.55) c	1.73(0.70) a	0.62(0.41) b	1.26(0.66) c

a, b, c: different letters mean significant differences between young and mature leaves and stems within each season and for each compound *p* < 0.05 (Wilcoxon Test). **Ap**: apigenin; **Ap-4′**: apigenin 4′-methyl ether; **Ap-7**: apigenin 7-methyl ether; **K-3**: kaempferol 3-methyl ether; **K-3.7**: kaempferol 3,7-di-*O*-methyl ether **D1**: 6-acetoxy-7-oxo-8-labden-15-oic acid; **D2**: 7-oxo-8-labden-15-oic acid; **D3**: oxocativic acid.

**Table 2:** Amount of flavonoids and diterpenes (standard deviation) in young leaves, mature leaves and stems of *C. ladanifer* in individuals of different ages (mg/g dw).

Compounds	Age	<1	2–6	7–11	12–16	17–21	22–26	27–31	>31
**Flavonoids (mg/g dw)**									
**Ap**	Young leaves	0.05 (0.01) a	0.10 (0.07) b	0.15 (0.09) c	0.09 (0.02) b,c	0.23 (0.07) c,d	0.30 (0.02) d	0.12 (0.04) b,c	0.09 (0.02) b,c
	Madures leaves		0.04 (0.02) a	0.06 (0.02) b	0.04 (0.01) a,b	0.06 (0.04) a,b	0.06 (0.01) a,b	0.06 (0.01) a,b	0.03 (0.00) a
	Stems		0.17 (0.07) a,b	0.17 (0.05) a	0.12 (0.06) b	0.15 (0.06) a,b	0.17 (0.01) a	0.19 (0.04) a	0.21 (0.01) a
**K-3**	Young leaves	1.64 (0.69) a	3.32 (1.50) b	3.66 (1.07) b	2.84 (0.90) b	4.78 (1.99) b,c	5.07(0.36) c	2.72 (0.78) b	2.23 (0.44) b,c
	Madures leaves		1.69 (0.68) a	2.02 (1.08) a	1.43 (0.31) a	2.13 (1.50) a	2.24 (0.22) a	1.85 (0.10) a	1.18 (0.32) a
	Stems		4.43 (1.41) a,b	5.25 (1.66) a,b	3.92 (2.07) a	4.68 (2.22) a,b	6.05 (0.42) b	4.91 (0.90) a,b	4.77 (0.76) a,b
**Ap-4′**	Young leaves	0.87 (0.39) a	1.24 (0.46) b	1.20 (0.30) b	1.29 (0.04) b	1.39 (0.77) a	1.99 (0.52) b	1.07(0.29) a,b	0.98 (0.09) a,b
	Madures leaves		0.75 (0.25) a	0.61 (0.07) a,b	0.64 (0.11) a,b	0.77 (0.32) a	0.77 (0.22) a	0.65 (0.08) a,b	0.54 (0.06) b
	Stems		1.77 (o.46) a	1.45 (0.20) b	1.50 (0.35) b	1.64 (0.32) a,b	2.20 (0.58) c	1.98 (0.39) a,c	1.60 (0.33) a,b
**Ap-7**	Young leaves	1.13 (0.52) a	1.83 (0.56) b	1.81 (0.46) b	1.39 (0.26) a	1.94 (0.97) a,b	2.20 (0.72) a,b	1.47 (0.17) a	1.50 (0.49) a,b
	Madures leaves		1.24 (0.44) a	1.16 (0.37) a	0.96(0.06) a	1.24 (0.47) a	1.29 (0.45) a	1.19 (0.15) a	0.88 (0.35) a
	Stems		1.51 (0.39 ) a	1.40 (0.13) a	1.27 (0.18) a	1.45 (0.21) a	1.67 (0.66) a	1.69 (0.02) a	1.50 (0.59) a
**K-3.7**	Young leaves	2.79 (1.58) a	1.67 (3.21) b	6.65 (1.40) b	6.44 (1.14) b	6.20 (1.78) b	5.59 (1.01) b	4.84 (0.54) b	4.07 (0.36) a,b
	Madures leaves		4.77 (1.09) a	5.64 (1.80) a	5.53 (1.86) a	5.17 (1.25) a	5.75 (0.98) a	5.09 (1.00) a	3.31 (0.59) b
	Stems		4.12 (1.39) a	5.26 (2.35) a	4.78 (2.14) a	4.00 (1.29) a	4.94 (0.89) a	5.22 (0.48) a	3.24 (0.89) a
**Diterpenes (mg/g dw)**									
**D1**	Young leaves	0.47 (0.43) a	0.80 (0.32) b	0.71 (0.25) a,b	0.87 (0.33) b	0.91 (0.68) b	1.50 (0.46) c	0.76 (0.12) a,b	0.41 (0.11) a
	Madures leaves		0.24 (0.14) a	0.14 (0.08) b,c	0.15 (0.05) a,b,c	0.22 (0.10) a,b	0.19 (0.05) a,b	0.22 (0.09) a,b	0.08 (0.02) c
	Stems		0.43 (0.15) a	0.42 (0.17) a,b	0.52 (0.31) a,b	0.64 (0.18) b	0.92 (0.36) c	0.80 (0.05) c	0.37 (0.13) a
**D2**	Young leaves	0.23 (0.19) a	0.58 (0.25) b	0.66 (0.18) b	0.63 (0.28) b	0.61 (0.62) a,b	1.07 (0.34) c	0.52 (0.01) b	0.43 (0.21) b
	Madures leaves		0.11 (0.05) a	0.09 (0.05) a,b	0.06 (0.03) b	0.09 (0.04) a,b	0.09 (0.03) a	0.12 (0.00) a	0.06 (0.00) b
	Stems		0.20 (0.11) a	0.38 (0.15) b	0.34 (0.10) b	0.38 (0.24) b	0.55 (0.31) c	0.45 (0.06) b,c	0.42 (0.12) b,c
**D3**	Young leaves	0.35 (0.17) a	0.88 (048) b	1.27 (0.74) b	0.66 (0.21) b	0.52 (0.15) a,b	0.74 (0.37) b	0.37 (0.16) a	0.70 (0.31) b
	Madures leaves		0.16 (0.09) a	0.16 (0.11) a,b	0.06 (0.02) b,c	0.12 (0.07) a,b	0.08 (0.03) b,c	0.07 (0.06) b,c	0.04 (0.00) c
	Stems		0.47 (0.23) a	1.01 (0.71) b	0.52 (0.28) a,b	0.55 (0.35) a,b	0.64 (0.25) a,b	0.57 (0.19) a,b	0.53 (0.24) a,b

a, b, c, d: different letters mean significant differences between ages for each compound *p* < 0.05 (Wilcoxon Test). **Ap**: apigenin; **Ap-4′**: apigenin 4′-methyl ether; **Ap-7**: apigenin 7-methyl ether; **K-3**: kaempferol 3-methyl ether; **K-3.7**: kaempferol 3,7-di-*O*-methyl ether; **D1**: 6-acetoxy-7-oxo-8-labden-15-oic acid; **D2**: 7-oxo-8-labden-15-oic acid; **D3**: oxocativic acid.

**Table 3:** Values for the population selected for sample collection: P: total seasonal rainfall (mm); Tmax: mean of the seasonal maximum temperatures (°C); Tmin: mean of the minimum seasonal temperatures (°C).

Climatic Parameters	Sprint	Summer	Autumn	Winter
P (mm)	46.7	11.8	359.4	155.7
Tmax (°C)	25.8	35.3	16.5	15.3
Tmin (°C)	11.4	17.4	8	5.2
